# Supplementary material for: Intermediate-Level Diet Quality of Brazilian Paralympic Athletes Based on National and International Indexes
Source: Nutrients. 2023 Jul 17;15(14):3163. doi: 10.3390/nu15143163 (PMC10384531; doi:10.3390/nu15143163)
Supplement: Supplementary file 1 [file nutrients-15-03163-s001.zip › nutrients-2454544-supplementary.pdf]

SUPPLEMENTARY MATERIALS

Table S1: Scoring criteria of Brazilian Healthy Eating Index Revised (BHEI-R) components.

BHEI-R components	Scoring range	Minimum score	Maximum score
Total Fruit *	0 to 5	No consumption	1.0 serving/1000kcal
Whole Fruit	0 to 5	No consumption	0.5 serving/1000kcal
Total Vegetables	0 to 5	No consumption	1.0 serving/1000kcal
Dark Green and Orange Vegetables **	0 to 5	No consumption	0.5 serving/1000kcal
Total Grains	0 to 5	No consumption	2.0 serving/1000kcal
Whole Grains	0 to 5	No consumption	1.0 serving/1000kcal
Milk and Dairy Products	0 to 10	No consumption	1.5 serving/1000kcal
Meat and Eggs **	0 to 10	No consumption	1.0 serving/1000kcal
Oils***	0 to 10	No consumption	0.5 serving/1000kcal
Saturated Fat	0 to 10	≥ 15% of TEI	≤ 7% of TEI
Sodium	0 to 10	≥ 2.0 g/ 1.000 kcal	≤ 0.75 g/ 1000 kcal
SoFAAS	0 to 20	≥ 35% of TEI	≤ 10% of TEI
Total BHEI-R	0 to 100		
Score: < 51 points “poor diet”, 51 to 80 points as “need modification”, > 80 points “healthy diet”			

* Represent the consumption of fruits as natural Juice

** Legumes added in this group

*** Include nuts and fish oil

TEI: Total Energy Intake; SoFAAS: solid fat, alcohol, and sugar added.

Table S2: Food groups and scoring method for the GDQS, the GDQS+, and the GDQS -

		GDQS components	Scoring range (g/day)				Respective point values			
Food Groups	GDQS +	Legumes	<9	9–42	>42		0	2	4	
		Nuts and seeds	<7	7–13	>13		0	2	4	
		Dark green leafy vegetables	<13	13–37	>37		0	2	4	
		Whole Grains	<8	8–13	>13		0	1	2	
		Liquid oils	<2	2–7.5	>7.5		0	1	2	
		Fish	<14	14–71	>71		0	1	2	
		Deep orange fruits	<25	25–113	>113		0	1	2	
		Citrus fruits	<24	24–69	>69		0	1	2	
		Other fruits	<27	27–107	>107		0	1	2	
		Low-fat dairy	<33	33–132	>132		0	1	2	
		Poultry	<16	16–44	>44		0	1	2	
		Eggs	<6	6–32	>32		0	1	2	
		Cruciferous vegetables	<13	13–36	>36		0	0.25	0.5	
		Deep orange vegetables	<9	9–36	>36		0	0.25	0.5	
		Deep orange tubers	<12	12–63	>63		0	0.25	0.5	
		Other vegetables	<23	23–114	>114		0	0.25	0.5	
	GDQS -	High-fat dairy	<35	35–142	142–734	>734	0	1	2	0
		Red meat	<9	9–46	>46		0	1	0	
		Juice	<36	36–144	>144		2	1	0	
		Processed Meat	<9	9–30	>30		2	1	0	
		White roots and tubers	<27	27–107	>107		2	1	0	
		Refined grains and baked goods	<7	7–33	>33		2	1	0	
		Sugar-sweetened beverages	<57	57–180	>180		2	1	0	
		Sweets and ice cream	<13	13–37	>37		2	1	0	
		Fried foods that are purchased	<9	9–45	>45		2	1	0	

Score: < 15 points - low risk; 15 to 23 points - moderate risk and > 23 points - high risk of noncommunicable disease

Metric scoring approach: For each food group, a point value is assigned based on the observed range of consumption in grams per day. For example, legume consumption <9g /day is assigned 0 points, 9–42 g/day is assigned 2 points, and >42 g/day is assigned 4 points. Each food group is scored using three scoring ranges except for high-fat dairy, which uses four ranges. The GDQS is obtained by summing all food group point values, the GDQS+ is obtained by summing only the point values for the subset of food groups whose point values increase with increasing consumption, and the GDQS- is obtained by summing only the point values for the subset of foods groups whose point values decrease with increasing consumption or (in the case of two food groups, red meat, and high-fat dairy) increase and then decrease after a specific consumption threshold is met.

Table S3: Descriptive scoring criteria of the usual intake and the first 24-hour recall of the Brazilian Healthy Eating Index Revised (BHEI-R) of 101 athletes with disabilities in Federal District/Brazil, 2018–2019

BHEI-R components	Usual Intake/Rec1	Median	IQR	Minimum	Maximum	p
Total Fruit	Usual Intake	2,46	2,26	0,37	4,32	0,03
	Rec1	1,16	5,00	0,00	5,00	
Whole Fruit	Usual Intake	2,20	2,40	0,35	4,14	0,04
	Rec1	0,00	4,09	0,00	5,00	
Total vegetables	Usual Intake	4,79	0,15	3,12	4,79	0,001
	Rec1	5,00	0,00	0,00	5,00	
Dark green and orange vegetables	Usual Intake	4,76	0,61	2,34	4,76	0,005
	Rec1	5,00	0,00	0,00	5,00	
Total grains	Usual Intake	4,05	0,65	2,52	4,65	
	Rec1	4,14	1,84	0,00	5,00	
Whole grains	Usual Intake	0,07	0,93	0,01	3,41	
	Rec1	0,00	0,97	0,00	5,00	
Milk and dairy products	Usual Intake	3,64	3,37	0,32	8,47	
	Rec1	2,62	5,61	0,00	10,00	
Meat and eggs	Usual Intake	9,73	0,24	7,80	9,73	0,001
	Rec1	10,00	0,00	0,22	10,00	
Oils	Usual Intake	9,67	0,44	5,08	9,67	0,001
	Rec1	10,00	0,00	0,00	10,00	
Saturared dat	Usual Intake	6,10	2,19	2,70	8,38	
	Rec1	7,15	6,32	0,00	10,00	
Sodium	Usual Intake	3,55	1,66	1,05	5,74	
	Rec1	3,15	4,68	0,00	10,00	
SoFAAS	Usual Intake	10,35	5,79	1,58	18,00	0,01
	Rec1	10,67	12,22	0,00	20,00	

SD = Standard Deviation; Rec1 = First 24-hour recall; Usual Intake = Calculation usual intake in software MSM with all 24-hour recalls; SoFAAS = energy from solid fat, alcohol, and added sugar.

Table S4: Descriptive scoring criteria of the usual intake and the first 24-hour recall of the Global Diet Quality Score (GDQS), GDQS Positive (GDQS +) and GDQS Negative (GDQS -) of 101 athletes with disabilities in Federal District/Brazil, 2018–2019

GDQS components	Usual Intake/Rec1	Median	IQR	Minimum	Maximum	p
Legumes	Usual Intake	2,68	1,00	1,17	3,21	0,001
	Rec1	4,00	2,00	0,00	4,00	
Nuts and Seeds	Usual Intake	0,00	0,92	0,00	3,05	
	Rec1	0,00	0,00	0,00	4,00	
Dark Green Leafy Vegetables	Usual Intake	0,53	0,90	0,04	2,74	
	Rec1	0,00	2,00	0,00	4,00	
Whole Grains	Usual Intake	0,10	0,72	0,07	1,41	
	Rec1	0,00	2,00	0,00	2,00	
Liquid Oils	Usual Intake	1,51	0,28	0,93	1,62	0,001
	Rec1	2,00	0,00	0,00	2,00	
Fish	Usual Intake	0,04	0,02	0,02	0,54	0,001
	Rec1	0,00	0,00	0,00	2,00	
Deep Orange Fruits	Usual Intake	0,00	0,00	0,00	1,52	
	Rec1	0,00	0,00	0,00	2,00	
Citrus Fruits	Usual Intake	0,03	0,01	0,01	0,50	0,001
	Rec1	0,00	0,00	0,00	2,00	
Other Fruits	Usual Intake	0,46	0,60	0,13	1,30	
	Rec1	0,00	1,00	0,00	2,00	
Low-fat Dairy	Usual Intake	0,00	0,00	0,00	1,60	
	Rec1	0,00	0,00	0,00	2,00	
Poultry	Usual Intake	0,49	0,88	0,04	1,70	
	Rec1	0,00	2,00	0,00	2,00	
Eggs	Usual Intake	0,55	0,51	0,14	1,44	0,009
	Rec1	1,00	2,00	0,00	2,00	
Cruciferous Vegetables	Usual Intake	0,00	0,00	0,00	0,39	
	Rec1	0,00	0,00	0,00	0,50	
Deep Orange Vegetables	Usual Intake	0,01	0,15	0,00	0,35	
	Rec1	0,00	0,25	0,00	0,50	
Deep Orange Tubers	Usual Intake	0,00	0,00	0,00	0,43	
	Rec1	0,00	0,00	0,00	0,50	
Other Vegetables	Usual Intake	0,13	0,15	0,03	0,36	0,001
	Rec1	0,25	0,50	0,00	0,50	
High-fat Dairy	Usual Intake	0,75	0,84	0,11	1,60	0,001
	Rec1	1,00	2,00	0,00	2,00	
Red Meat	Usual Intake	-	-	-	-	€
	Rec1	0,00	0,00	0,00	1,00	
Juice	Usual Intake	1,49	0,84	0,35	1,92	

	Rec1	2,00	2,00	0,00	2,00	
Processed Meat	Usual Intake	1,55	0,54	0,51	1,85	
	Rec1	2,00	1,00	0,00	2,00	
White Roots and Tubers	Usual Intake	1,65	0,29	0,62	1,66	
	Rec1	2,00	1,00	0,00	2,00	
Refined Grains and Baked Goods	Usual Intake	0,52	0,47	0,22	1,32	0,001
	Rec1	0,00	0,00	0,00	2,00	
Sugar-sweetened Beverages	Usual Intake	1,99	0,51	0,28	1,99	0,004
	Rec1	2,00	0,00	0,00	2,00	
Sweets and Ice Cream	Usual Intake	1,09	0,74	0,27	1,70	0,001
	Rec1	1,00	2,00	0,00	2,00	
Fried Foods that are Purchased	Usual Intake	1,97	0,00	1,97	2,00	€
	Rec1	2,00	0,00	2,00	2,00	
GDQS -	Usual Intake	9,63	1,42	7,20	12,31	
	Rec1	10,00	4,00	2,00	15,00	
GDQS +	Usual Intake	11,17	3,09	5,77	19,55	
	Rec1	10,25	5,50	0,00	23,25	0,001

SD = Standard Deviation; Rec1 = First 24-hour recall; Usual Intake = Calculation of usual intake with software MSM with all 24-hour recalls; € = Not possible to apply the Wilcoxon teste because the distribution asymmetry.

Table S5: Socio-demographics and sport related support presented as number and the sum of rank from 101 athletes with disabilities from 13 Paralympic sports stratified by the Global Diet Quality Score (GDQS) according to the usual intake or the first 24-hour recall (Rec1) - Federal District/Brazil, 2018–2019

Characteristics	Rec1 / Usual	Groups	n	Rank	p
Sex	Usual	Men	82	4090,00	0,42
		Women	19	1061,00	
	Rec1	Men	82	4077,50	0,36
		Women	19	1073,50	
Sport	Usual	Individual	45	2431,00	
		Team	56	2720,00	0,35
	Rec1	Individual	45	2552,50	
		Team	56	2598,50	0,08
Age	Usual	18-30y	37	1723,00	
		30y+	64	3428,00	0,25
	Rec1	18-30y	37	1627,00	
		30y+	64	3524,00	0,07
Income	Usual	Low	67	3430,00	0,93
		High	34	1721,00	
	Rec1	Low	67	3377,50	0,78
		High	34	1773,50	
Sports scholarship	Usual	No	55	2618,00	0,20
		Yes	46	2533,00	
	Rec1	No	55	2738,50	0,65
		Yes	46	2412,50	
Nutritional support	Usual	No	70	1814,00	
		Yes	31	3337,00	0,09
	Rec1	No	70	2051,50	
		Yes	31	3099,50	0,00
Education	Usual†	Primary education	24	-	
		Secondary education	43	-	
		Tertiary education or equivalent	34	-	
	Rec1	Primary education	24	55,42	
		Secondary education	43	41,58	
		Tertiary education or equivalent	34	59,79	0,02*
Ranking Level	Usual	International	23	1258,00	
		Regional / National	78	3893,00	0,49
	Rec1	International	23	1305,50	
		Regional / National	78	3845,50	0,28

* p adj. - The difference was “tertiary education or equivalent” had a higher score than to “secondary education” from Kruskal–Wallis test followed by the Bonferroni-Dunn post hoc. All remaining analysis with Mann Whitney Test. † Kruskal–Wallis test did not converge.

Table S6: Socio-demographics and sport related support presented as number and the sum of rank from 101 athletes with disabilities from 13 Paralympic sports stratified by the Brazilian Healthy Eating Index Revised (BHEI-R) according to the usual intake or the first 24-hour recall (Rec1) - Federal District/Brazil, 2018–2019

Characteristics	Rec1 / Usual	Groups	n	Rank	p
Sex	Usual	Men	82	4133,00	0,67
		Women	19	1018,00	
	Rec1	Men	82	4071,00	0,33
		Women	19	1080,00	
Sport	Usual	Individual	45	2545,00	
		Team	56	2606,00	0,09
	Rec1	Individual	45	2631,00	0,02
		Team	56	2520,00	
Age	Usual	18-30y	37	1763,00	
		30y+	64	3388,00	0,38
	Rec1	18-30y	37	1973,00	
		30y+	64	3178,00	0,54
Income	Usual	Low	67	3402,00	0,91
		High	34	1749,00	
	Rec1	Low	67	3417,00	1,00
		High	34	1734,00	
Sports scholarship	Usual	No	55	2577,00	0,12
		Yes	46	2574,00	
	Rec1	No	55	2647,00	0,28
		Yes	46	2504,00	
Nutritional support	Usual	No	70	1920,00	
		Yes	31	3231,00	0,01
	Rec1	No	70	1875,00	
		Yes	31	3276,00	0,03
Education†	Usual	Primary education	24	-	
		Secondary education	43	-	
		Tertiary education or equivalent	34	-	
	Rec1	Primary education	24	-	
		Secondary education	43	-	
		Tertiary education or equivalent	34	-	
Ranking Level	Usual	International	23	1342,00	
		Regional / National	78	3809,00	0,17
	Rec1	International	23	1254,00	
		Regional / National	78	3897,00	0,51

Mann Whitney Test was used. † Kruskal–Wallis test did not converge.
